# Multiple Biomarker-Combined Screening for Colorectal Cancer Based on Bisulfate Conversion-Free Detection of Fecal DNA Methylation

**DOI:** 10.1155/2021/1479748

**Published:** 2021-09-28

**Authors:** Chong Liu, Lei Xu, Wei Li, Min Jie, Wei Xue, Wenqiang Yu

**Affiliations:** ^1^Department of Gastroenterology, Hunan Aerospace Hospital, Changsha, Hunan Province, China 410005; ^2^Department of Gastroenterology, People's Hospital of Ningxiang, Changsha, Hunan Province, China 410600

## Abstract

To evaluate the applicability of bisulfate conversion-free methylation assay based on enzyme digestion in fecal screening for colorectal cancer (CRC). Stool samples were collected from a total of 1142 participants with intestinal abnormalities, including 180 positive cases, 60 advanced adenomas, and 902 negative cases. DNA from reference cell lines and clinical samples was extracted and digested with an enzyme to detect the methylation of CRC markers SEPT9, SDC2, NDRG4, SFRP2, and BMP3 genes. Statistical analysis was then used to determine the ability of the markers, both individually and in combination, to detect CRC and adenoma. Our results showed that the enzyme digestion method could suitably detect DNA marker methylation in as low as 1% of the cell lines. BMP3 had a considerably low detection rate in all clinical samples, with only 6 positive cases detected out of 180 cancer samples. Our findings showed that the combination of SEPT9, SDC2, and SFRP2 had an area under the receiver operation curve of 0.937, sensitivity of 94.11%, and specificity of 89.21% for detecting CRC. Moreover, the detection sensitivity of adenoma can also reach 38.33%. After innovatively utilizing bisulfate conversion-free methylation assay for CRC screening, this study verified the potential clinical applicability of combining multiple biomarkers for CRC screening in a large number of samples.

## 1. Introduction

Colorectal cancer (CRC), the third most common type of malignancy in Western countries, has continued to rapidly increase in developing countries [1–3]. Worldwide estimates have shown that approximately 1.85 million individuals had been diagnosed with CRC and approximately 880,000 had died from the same in 2018 [4]. Owing to the popularization of CRC screening, the incidence of CRC in developed countries has decreased to a certain extent, although we have also witnessed a trend of younger patients developing CRC [5]. CRC differs from other malignancies, such as lung and liver cancers, in that earlier CRC detection promotes better survival rates. Large-scale implementation of simple CRC screening could significantly increase early detection of tumors and improve survival rate [6, 7].

Colonoscopy has remained the gold standard method for the clinical screening of CRC despite being an invasive procedure with certain risks, such as bleeding and anesthesia, troublesome pretreatment, and some contraindications, causing low acceptance among patients [8–10]. Noninvasive detection methods, such as fecal immunochemical tests for hemoglobin and imaging, have displayed low sensitivity and specificity, which could easily cause false positive or false negative results especially in the detection of colorectal adenomas or stage I CRCs [11–13]. Therefore, there is an urgent need for a noninvasive, convenient, rapid, sensitive, and specific screening method for CRC and its precancerous lesions.

Recently, several noninvasive gene detection methods have emerged, such as the detection of septin 9 gene methylation in the blood [14, 15] and syndecan 2 (SDC2) gene methylation in stool cell-free DNA (cfDNA) that have been commercially detected [16]. Numerous studies have also found that N-Myc downstream-regulated gene 4 protein (NDRG4) [17], secreted frizzled-related protein 2 (SFRP2) [1], secreted frizzled-related protein 1 (SFRP1) [18], and vimentin [19, 20] are associated with the occurrence of CRC. Evidence has shown that the Chinese population has a substantially low mutation rate in the marker bone morphogenetic protein 3 (BMP3) of Cologuard, a star product included in medical insurance in the United States, making it an unsuitable screening marker [21].

cfDNA from cancer tissue is barely present in either feces or blood, with its detection often requiring a highly sensitive assay. However, traditional methylation detection methods usually employ bisulfate treatment, which discards a considerable portion of the DNA template and has a cumbersome and time-consuming process. The enzyme digestion method has been adopted for the enrichment and detection of methylated fragments given its simplicity, shorter processing time, and improved DNA utilization, which is conducive for the detection of trace cfDNA molecules in stool or blood. The current study evaluated the potential applicability of enzyme digestion in detecting methylation in stool samples from patients with CRC, presenting a novel method for detecting methylation among such patients.

## 2. Materials and Methods

### 2.1. Sample Collection

A total of 1142 samples were collected from Hunan Aerospace Hospital and People's Hospital of Ningxiang in the central and southern region of China, respectively, and initially diagnosed with recurrent diarrhea, abdominal pain, anemia of unknown cause, and other intestinal clinical symptoms. A 50 mL sample collection tube containing 10 mL of buffer was then used to collect approximately 5 g of fecal samples from subjects, after which they were shaken well. Samples were delivered to the laboratory within 4 h and refrigerated at −20°C for subsequent DNA extraction.

The clinical symptoms, colonoscopy results, and pathological test results of the subjects were recorded in detail, with relevant clinical information regarding the samples being summarized in Table 1 (180 cases of pathologically confirmed CRC, 60 cases of adenoma, 273 cases of polyps, and 629 cases of inflammation and normal samples).

### 2.2. DNA Isolation

Stool samples were homogenized in preservative buffer using a shaker device. After homogenization, the sample was centrifuged at 4000g for 15 min, after which 10 mL of supernatant was transferred into a new tube. Thereafter, 10 mL of lysis buffer (GenMagBio, Beijing) was added into the supernatant followed by incubation at 55°C for 20 min. Afterwards, 2 mL of 10% polyvinylpolypyrrolidone was added followed by incubation with a mixer at room temperature for 30 min. The sample was then centrifuged at 4000g for 15 min, subsequently transferring the supernatant into a new tube. Thereafter, 30 *μ*L of Acryl Carrier, 60 *μ*L of magnetic beads (GenMagBio, Beijing), and 240 *μ*L of proteinase K were added into this tube followed by incubation at room temperature for 30 min. The tube was then placed on a magnet until the solution cleared, and the beads were pelleted against the magnet. After discarding the supernatant, the beads were washed with wash buffer twice and then eluted with 60 *μ*L of distilled water. Thereafter, DNA concentrations were measured using NanoDrop 2000 (Thermo Scientific, MA, USA). All purified DNA was stored at −20°C.

The feasibility of the enzyme digestion test scheme was confirmed by establishing the standard product test system. To simulate a stool sample, the HCT116 cell line (Yinrun, Changsha), with known fully methylated septin 9 (SEPT9) gene, and TE10 cell line (Yinrun, Changsha), with known fully methylated NDRG4 gene, were mixed with the stool of a normal person who had undergone colonoscopy, subsequently extracting the DNA using the aforementioned process. Positive reference DNA was extracted, and its concentration was determined using NanoDrop, which should be at 1.8 ≤ OD260/280 ≤ 2.0. To ensure that a lower percentage of mutated DNA can be detected, a commercial simulated cfDNA (Invitrogen, Shanghai) was added to the aforementioned DNA at a concentration of 10 ng/*μ*L, after which the proportion of cell-line DNA was adjusted to 0.5%, 1%, and 5%, respectively. Normal saline containing 0.9% sodium chloride was used as a negative quality control substance in this study.

### 2.3. Enzyme Digestion Process

A total of 10 *μ*L (500 ng) of extracted and purified DNA samples was digested using 10x buffer diluted five times with restriction enzyme BstUI (Thermo Scientific) and incubated at 60°C for 1 h. After enzymatic digestion, the nucleic acid did not need to be purified, with the DNA concentration after digestion being 10 ng/*μ*L. The treated DNA was used immediately or stored at −20°C. Positive reference samples were treated in the same manner as clinical samples to be tested.

### 2.4. Quantitative Methylation-Specific Polymerase Chain Reaction (qMSP)

#### 2.4.1. Primer Design

**Figure 1:**
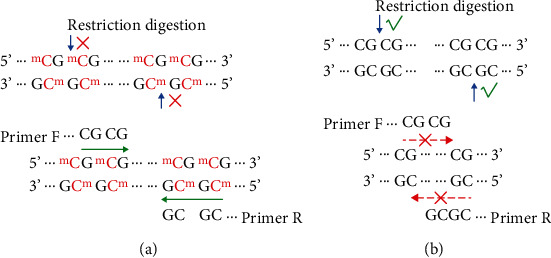
Schematic diagram of bisulfate-free methylation detection: (a) methylated sequence; (b) nonmethylated sequence.

Fluoresome polymerase chain reaction (PCR) primers (Table 2) and probe primers (Table 3) were designed for target genes SEPT9, BMP3, SFRP2, NDRG4, and SDC2, as well as the reference gene actin beta (ACTB). Notably, it was necessary to ensure that the 3′ end of the upstream and downstream primers of the target gene primer was CGC or CGCG. Moreover, the number of “CGCG” loci contained in the primer sequence can be selected according to the specific template sequence, with more “CGCG” loci in the preferred primer leading to higher specificity. The detection schematic diagram is shown in Figure 1.

#### 2.4.2. PCR Amplification

After diluting the 10 ng/*μ*L sample from the previous steps to 2 ng/*μ*L, 2 *μ*L Taq enzyme was added into the reaction tube. The concentration of primers for each gene was 0.15 *μ*m, the concentration of the probe primers was 0.1 *μ*m, and the concentration of tetramethylammonium chloride was 35 mM, forming a reaction system of 30 *μ*L in total. The whole tube was initially reacted at 95°C for 5 min, followed by 5 cycles of 95°C for 15 s and 60°C for 30 s. Thereafter, 35 cycles of amplification are done at 95°C for 15 s and 63°C for 30 s. Finally, the tubes were incubated at 20°C for 2 min. The results were then derived of the instrument, and the CT values of each detection marker were obtained.

### 2.5. Statistical Analysis

The Wilcoxon rank sum test was used to compare methylation levels between different groups. Receiver operation characteristic (ROC) curve and area under the ROC curve (AUC) values were estimated to evaluate diagnostic accuracy, while Delong's test was used to compare differences in AUC values. The model effect and coefficients were plotted using jtools. All statistical analyses were conducted using R version 3.5.0., with *P* < 0.05 indicating statistical significance. A logistic model was created using the general linear model (glm) function: *x* = *h* + *k*1∗ CT value of target gene 1 + ⋯+*kn*∗ CT value of target gene *n*, subsequently calculating the model value *x*. Thereafter, the model value *x* was substituted into the sigmoid function *f*(*x*) = 1/(1 + *e*^−*x*^ ) to obtain a fit value, *f*(*x*), for diagnostic purposes.

## 3. Results

### 3.1. Detection of Methylation Efficiency Using Enzyme Digestion

**Figure 2:**
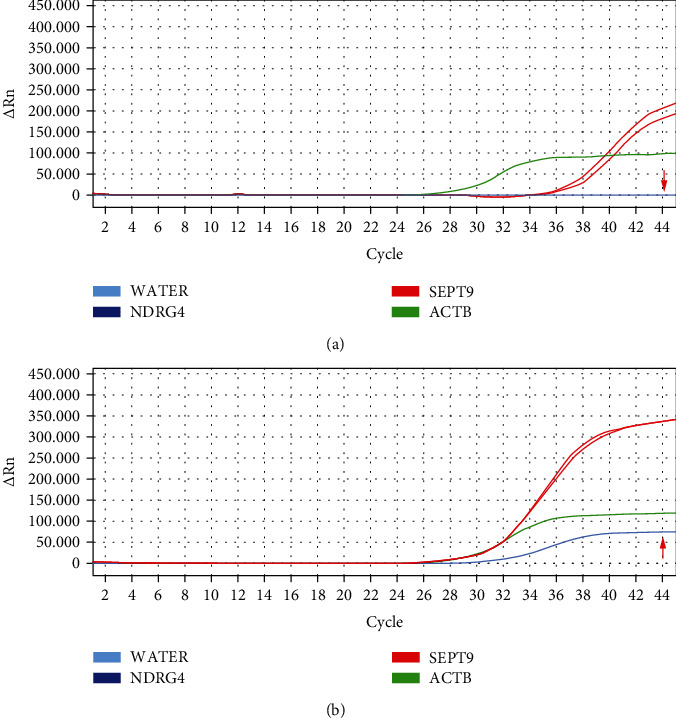
Polymerase chain reaction amplification curve with (a) and without (b) enzyme treatment.

The methylation efficiency of the enzyme digestion method was confirmed by detecting cell-line DNA in the stool. Accordingly, enzyme digestion promoted higher purity of the methylated fragment and earlier appearance of the change in peak value compared to its absence (Figure 2(a)). Meanwhile, the absence of enzymatic digestion caused some negative references to peak at the late stage, resulting in instability (Figure 2(b)). The aforementioned results showed that the positive reference could be detected stably at a methylated DNA concentration of ≥1% (Table 4), after three repeated tests with varying positive reference DNA concentrations. Moreover, our results showed that our enzyme digestion method could detect cfDNA methylation in stool.

**Figure 3:**
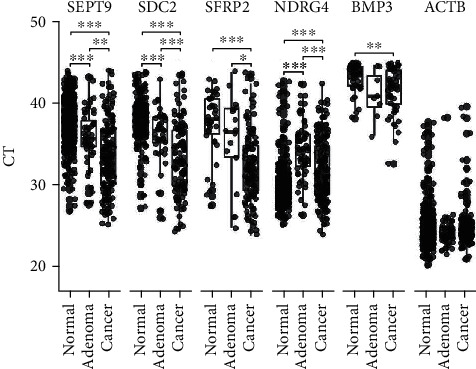
The distribution of amplified computed tomography values of each gene in different samples.

After evaluating a large number of samples, our results showed that all samples had good detection efficacy across different markers, while the CT value of the reference gene ACTB was within a reasonable range (Figure 3), indicating a high pass rate.

### 3.2. Comparison of Detection Rates among All Markers across Different Clinical Manifestations

**Figure 4:**
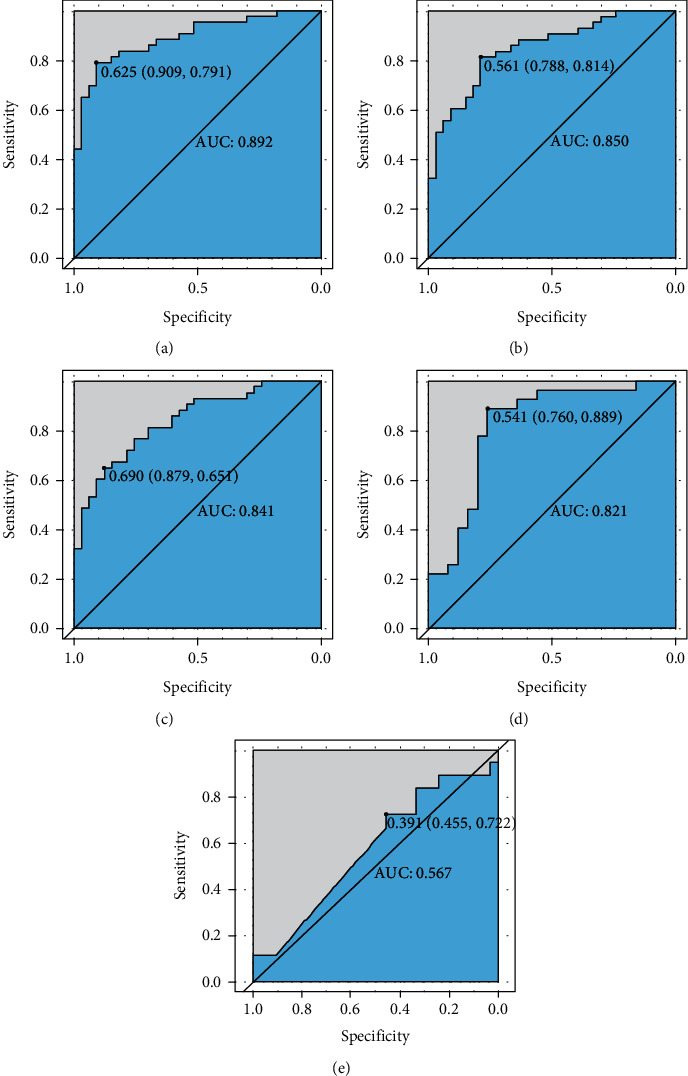
Performance of each gene for colorectal cancer screening. (a) Receiver operating characteristic (ROC) curve of SDC2; (b) ROC curve of SFRP2; (c) ROC curve of SEPT9; (d) ROC curve of NDRG4; (e) ROC curve of BMP3.

All samples were tested for methylation. Positivity rates of cfDNA target genes in stool samples from normal individuals with different clinical manifestations, including cancer, adenoma, polyp, and normal samples, are shown in Table 5. Accordingly, we found that BMP3 had considerably low positivity rates in all groups, while other markers had a high positive rate in those with malignant tumors and adenomas and had good specificity in those with polyps and control samples. Our results showed that SEPT9 had the best sensitivity (88.33%) but low specificity (87.12%). Meanwhile, SDC2 and SFRP2 had high specificity, reaching 96.7% and 98.17%, respectively, but slightly lower sensitivity compared to SEPT9. Moreover, our results showed that a sensitivity of 94.44% was achieved when positive methylation of at least one of the five markers was considered as a high risk for CRC. Notably, sensitivity for adenoma detection was 38.33%, while specificity for polyp detection and normal samples was 79.49% and 86.65%, respectively. The AUC of each marker for predicting CRC is presented in Figure 4. Accordingly, SDC2 had the highest AUC at 0.892, whereas BMP3 had the lowest AUC at only 0.567.

### 3.3. Combination of Different Markers for Positive Prediction

60% of the various types of samples (108 cases of pathologically confirmed CRC, 36 cases of adenoma, 164 cases of polyps, and 378 cases of inflammation and normal samples) were randomly selected to form the training set, and the remaining 40% of each group of samples were randomly divided into two parts. 20% of the samples were used as the validation set for the selection of the model, and the last 20% of the samples were used to test the performance of the model in predicting CRC.

**Figure 5:**
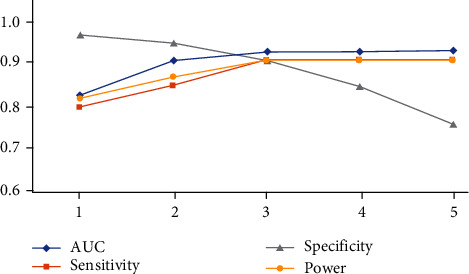
Prediction of colorectal cancer in the context of the cumulative effect of multiple markers. The number on the *X*-axis represents the number of markers used by the model, and the markers that are selected in the front after the model are ranked according to the importance of each marker.

**Figure 6:**
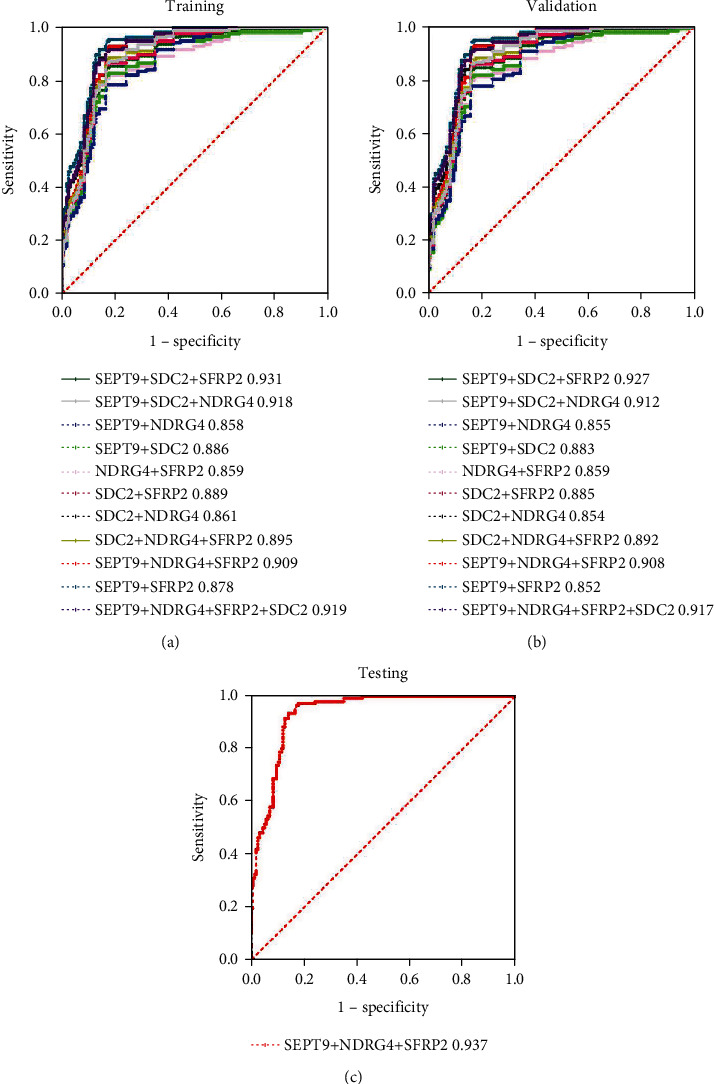
Colorectal cancer screening performance according to the combination of multiple genes: (a) training; (b) validation; (c) testing.

With each marker as an independent factor influencing the results, models were used to comprehensively evaluate the cumulative effect in CRC prediction, and the results showed that AUC and sensitivity reached their maximum when three markers, SEPT9, SDC2, and SFRP2, were used (Figure 5). Further analysis of the predicted CRC performance of the various combinations, with the retention of the combinations with AUC greater than 0.85, showed that the combined SEPT9, SDC2, and SFRP2 achieved good performance for predicting CRC (Figure 6(a)). Then, we randomly selected half of the remaining samples for verification, and the results showed that the data of each marker combination showed that the training model was relatively stable (Figure 6(b)). Finally, the remaining samples were used to verify the best marker combination in the previous model, and it is found that the CRC prediction performance is very good (Figure 6(c)). Accordingly, BMP3 was not included in the analysis given its substantially low positivity rate and inadequate AUC for use as a screening marker. Our results showed that the combination of SEPT9, SDC2, and SFRP2 achieved good performance for predicting CRC, with an AUC of 0.937 and a sensitivity and specificity of 94.11% and 89.21%, respectively. It is worth noting that validation of findings in independent samples would be needed to assess potential clinical utility.

### 3.4. Prediction Model Comprising Optimal Biomarkers for CRC

Finally, through the above data analysis, we obtained the parameters related to CRC risk assessment of the best model: *x* = 15.72465 − 0.04831∗CT (SEPT9) − 0.02987∗CT (SDC2) − 0.07796∗CT (SFRP2) − 0.005213∗CT (ACTB). The value corresponding to the maximum value of the Youden index on the ROC curve was taken as the threshold value. Accordingly, *f*(*x*) > 0.2297426 suggests malignancy, whereas *f*(*x*) < 0.2297426 indicates a benign state.

In summary, our findings showed that the enzyme digestion method had good ability for detecting methylation in stool samples. After analyzing a large number of samples, our results found that the combination of SEPT9, SDC2, and SFRP2 had good detection performance, making it a potential clinical screening method for CRCs.

## 4. Discussion

Commercially available fecal DNA methylation analyses have been used for CRC screening due to their noninvasive and highly sensitive characteristics. For instance, the United States Food and Drug Administration had approved the use of Cologuard®, whereas the Chinese National Medical Products Administration had approved the use of SDC2 [22]. Cologuard® detects KRAS gene hot spot mutations, as well as BMP3 and NDRG4 methylation, and comprehensively evaluates CRC risk with a sensitivity and specificity of 92% and 87%, respectively [23]. The current study utilized popular commercially available targets and markers frequently reported in the literature in a large number of clinical samples from multiple centers to determine the possibility of combining multiple CRC markers in a Chinese population. Accordingly, our findings showed that the combination SEPT9/SDC2/SFRP2 exhibited the best detection performance, with an AUC reaching 0.947. Simultaneously, the current study found that BMP3 had poor ability to detect CRC in the Chinese population, similar to that published in a previous study [21], with an AUC of only 0.567. Moreover, we found that these markers also played a role in adenoma screening, with a sensitivity of 38.33% for adenoma detection.

Traditional methylation detection has mainly utilized traditional bisulfite conversion, which converts unmethylated cytosine (C) in nucleic acid to uracil (U), while the methylated cytosine remains unchanged. An obvious sequence difference between methylated and unmethylated CpG islands can be observed after transformation [24]. The design of methylation-specific primers for fluorescence quantitative PCR has allowed the detection of target gene methylation in the sample nucleic acid with high specificity. However, bisulfite conversion causes serious DNA fragmentation, while purification promotes large amounts of DNA loss, which considerably affect detection sensitivity and result in false-negative test findings. Based on actual laboratory testing, this entire process usually requires more than 36 h, with cumbersome testing steps. Moreover, given the impossibility of automation, substantial manual operation would be required.

According to our laboratory examination, cfDNA in the feces of patients with CRC was largely derived from a large amount of microbial degradation, with very low proportions of DNA from CRC cells [25, 26]. Therefore, a highly sensitive technique or that with good target DNA enrichment would be required for detecting tumor-derived mutated DNA. BstUI is a restriction enzyme that can specifically recognize CGCG sites. Under appropriate conditions, the enzyme cleans CGCG sites from guanine (G) to cytosine (C). Moreover, the enzyme is sensitive to methylation and cannot recognize methylated CGCG sites [27, 28]. The specificity of BstUI restriction endonuclease site recognition was used to digest the nucleic acid, and specific primers were designed for fluorescence quantitative PCR detection to improve detection sensitivity.

Bisulfate conversion-free DNA need not undergo a robust purification process and can be directly used for PCR detection [29]. This leads to higher DNA recovery rates, thereby increasing sensitivity for detecting early-stage tumors, such as adenomas. Our results showed that when the cell lines were mixed into a stool sample, methylation levels in as low as 1% of the target DNA were consistently detected. Furthermore, the entire testing process had been completed within only 6 h. Compared to the traditional method, our method could save time and be less cumbersome, while also reducing the cost owing to manual operation.

The current study established a bisulfate-free methylation detection system by detecting cell lines to achieve highly sensitive detection of fecal DNA methylation. Through the detection of 1142 clinical samples, our results found that combining the methylation levels of three markers (i.e., SEPT9, SDC2, and SFRP2) achieved good performance for predicting CRC, suggesting its potential for clinical application.

## Figures and Tables

**Table 1:** Characteristics of the patients enrolled in the study.

	CRC	Adenoma	Polyp	Normal	Total
No. of participants	180	60	273	629	1142
Male sex (%)	577 (50.52%)				
Age range (average, yr)	26-83 (49.2)				
Tumor location					
Right	126				
Left	54				
Stage					
I–II	67				
III–IV	113				

**Table 2:** Primers for PCR amplification.

Genes	Primers
SEPT9-F	5′-GTCGGATTTCGCGGTTAACGC-3′
SEPT9-R	5′-CAACCAACCCAACACCCACCTT-3′
BMP3-F	5′-TTTGAAAATATTCGGGTTATATACGTCGC-3′
BMP3-R	5′-ATAAACTCTTCCCCAACAACTACGCGAA-3′
SFRP2-F	5′-CGGAGCCCCCCGGAGCTGCGC-3′
SFRP2-R	5′-TGGCAGCCGGCGGCTGGGGCGC-3′
SDC2-F	5′-AGGAGGAGGGGCGCAGCCGC-3′
SDC2-R	5′-GCAGAGCGGCGGGAGCGC-3′
NDRG4-F	5′-GGGTGTCCCCCAGGCTCCGC-3′
NDRG4-R	5′-GTGGCTTCCGCCTTCTGCGC-3′
ACTB-F	5′-GATGACCCAGGTGAGTGGCCCGCTACCTC-3′
ACTB-R	5′-GAGAGAACCAGTGAGAAAGGACGCAG-3′

**Table 3:** Fluorescent probe primers.

Genes	Primers
SFRP2	FAM-5′-CTTGCAGCGCCTCGCCCGCGCTGT-3′-BHQ2
SDC2	CY5-5′-AGCCAGTGGCCCCGCTTGGACG-3′-BHQ2
NDRG4	ROX-5′-CGCGGTCCCCGCTCGCCCTCCCGC-3′-BHQ2
SEPT9	P2-5′-TAGTTGGATGGGATTATTTCGGATTTCG-3′-BHQ2
BMP3	C5P1-5′-AGCGTTGGAGTGGAGACGGCGTTCGTAGCGT-3′-BHQ2
ACTB	HEX-5′-TCTGGTGGCCGCCTCCCTCCTTCCTGGCCTC-3′-BHQ2

**Table 4:** CT values of markers in reference cell lines detected by bisulfite-free methods.

Genes	Reference DNA concentration	CT (test 1)	CT (test 2)	CT (test 3)
SEPT9	5%	36.46	36.89	37.21
	1%	42.09	41.52	41.33
	0.5%	44.01	—	—
NDRG4	5%	37.92	38.96	37.61
	1%	41.37	41.98	42.18
	0.5%	—	—	44.08
ACTB	5%	31.87	32.14	33.66
	1%	35.53	35.01	36.47
	0.5%	41.99	42.05	42.36

**Table 5:** Sensitivity and specificity of methylated stool DNA among subgroups.

Different groups		SDC2	SEPT9	SFRP2	NDRG4	BMP3	Combination^#^
CRC (*n* = 180)	No. of positive results	116	159	106	124	6	170
	Sensitivity (%)	64.44%	88.33%	58.89%	68.89%	3.33%	94.44%
Adenoma (*n* = 60)	No. of positive results	11	21	15	23	1	23
	Sensitivity (%)	18.33%	35%	25%	38.33%	1.66%	38.33%
Polyp (*n* = 273)	No. of positive results	9	41	5	55	1	56
	Specificity (%)	96.7%	84.98%	98.17%	79.85%	99.63%	79.49%
Normal (*n* = 629)	No. of positive results	25	81	20	83	2	84
	Specificity (%)	96.03%	87.12%	96.82%	86.8%	99.68%	86.65%

^#^Combination: positive methylation of at least one of the five markers (five markers were used in the model: SFRP2 + SDC2 + NDRG4 + SEPT9 + BMP3) was considered high risk for CRC.

## Data Availability

The datasets used and/or analyzed during the current study are available from the corresponding author on reasonable request.

## Abbreviations

CRC: Colorectal cancer
PCR: Polymerase chain reaction
SDC2: Syndecan 2
SEPT9: Septin 9
cfDNA: Cell-free DNA
NDRG4: N-Myc downstream-regulated gene 4 protein
SFRP2: Secreted frizzled-related protein 2
SFRP1: Secreted frizzled-related protein 1
BMP3: Bone morphogenetic protein 3
ACTB: Actin beta
TMAC: Tetramethylammonium chloride
ROC: Receiver operation curve
KRAS: Cellular transforming protooncogene
AUC: The area under the curve
FDA: Food and Drug Administration
NMPA: National Medical Products Administration.
